# Health system stress: Assessing the impact of the COVID-19 pandemic on obstetric haemorrhage-related mortality in South Africa using confidential enquiry into maternal deaths data

**DOI:** 10.4102/safp.v68i1.6259

**Published:** 2026-04-23

**Authors:** Gomolemo Rakale, Sam T. Ntuli, Tshepo Ramarumo, Solly M. Seeletse

**Affiliations:** 1 Department of Statistical Sciences, Faculty of Science and Technology, Sefako Makgatho Health Sciences University, Pretoria, South Africa

**Keywords:** maternal mortality, obstetric haemorrhage, COVID-19 pandemic, health system resilience, South Africa

## Abstract

**Background:**

The coronavirus disease 2019 (COVID-19) pandemic placed unprecedented strain on healthcare systems worldwide, potentially exacerbating existing vulnerabilities in maternal healthcare. This study examines the pandemic’s impact on obstetric haemorrhage-related mortality in South Africa using the data from the Confidential Enquiry into Maternal Deaths (CEMD).

**Methods:**

We conducted a retrospective analysis of maternal mortality data from 2017–2023, focusing on obstetric haemorrhage mortality trends before, during and after the COVID-19 pandemic. Data were extracted from national confidential enquiries, including institutional maternal mortality ratios (iMMR) and absolute death numbers.

**Results:**

The analysis revealed a significant increase in obstetric haemorrhage mortality during the peak pandemic years (2020–2021), with deaths rising from 179 in 2019 to 237 in 2021, and iMMR increasing from 18.1 to 23.3 per 100 000 live births. This was followed by a substantial decline in subsequent years (2022–2023). The findings suggest that initial pandemic disruptions severely impacted maternity care services, while subsequent recovery reflects adaptive strategies and resource reallocation.

**Conclusion:**

The COVID-19 pandemic exposed critical vulnerabilities in South Africa’s ability to maintain essential maternity services during health system shocks.

**Contribution:**

The confidential enquiry process provides invaluable insights for strengthening health system resilience and preparing for future emergencies.

## Introduction

Obstetric haemorrhage, predominantly postpartum haemorrhage (PPH), persists as a leading cause of maternal mortality globally, representing a critical challenge to global health equity.^1,2^

In South Africa (SA), despite concerted national efforts and robust maternal death surveillance systems like the Confidential Enquiry into Maternal Deaths (CEMD),^3^ preventable deaths from haemorrhage persist as a significant public health concern.^4,5^ Maintaining advances in lowering the maternal mortality ratio (MMR) is challenging, making it susceptible to health system weaknesses. The coronavirus disease 2019 (COVID-19) pandemic, caused by a novel coronavirus strain first identified in Wuhan, China, was declared a global health emergency by the World Health Organization (WHO) on 11 March 2020.^6^ This pandemic significantly disrupted health systems and damaged critical health services globally.^7,8^ The reallocation of financial, human, and critical care resources, coupled with disruptions in supply chains and patient access to care, threatened to undermine decades of progress in maternal health outcomes.

## Research methods and design

### Study design

This study employed a retrospective analytical design using secondary data from the CEMD in SA. The confidential enquiry process involves a systematic, multidisciplinary review of the care received by women who died during pregnancy or within 42 days of termination of pregnancy, irrespective of the cause of death. This approach allows for both the quantification of mortality trends and the qualitative assessment of avoidable factors and quality of care issues.

### Study population

The study population consists of all maternal deaths as a result of obstetric haemorrhage that occurred in SA during the defined study period (2017–2023).

### Sample size and sampling technique

The investigation utilised a comprehensive census approach, incorporating all identified and officially documented instances of maternal deaths attributable to obstetric haemorrhage across SA during the specified study period.

### Data sources

The primary data source was the national aggregate data on obstetric haemorrhage mortality compiled by the CEMD for the years 2017–2023. This included absolute numbers of deaths and institutional maternal mortality ratios (iMMR) per 100 000 live births. The confidential enquiry methodology used in SA is a structured process where each maternal death is reviewed by a multidisciplinary committee consisting of experienced personnel representing obstetrics, midwifery, anesthesia and provincial health departments. The process includes identification of deaths through facility registers and death notification systems, conducting detailed case note reviews, assessing avoidable factors, and formulating recommendations for quality improvement.

### Data analysis

This study analysed national obstetric haemorrhage mortality trends from 2017 to 2023, with a specific focus on the pandemic’s impact by comparing pre-pandemic (2017–2019), peak-pandemic (2020–2021) and post-peak (2022–2023) periods. Given that CEMD provincial data are formally structured and interpreted in triennial cycles, we compared the periods 2017–2019 and 2020–2022 to assess subnational variations. The statistical analysis was conducted with STATA 16.0 software (StataCorp; College Station, Texas, United States).

### Ethical considerations

Ethical approval was secured prior to data collection from the Research Ethics Committee of Sefako Makgatho Health Sciences University (Reference number: SMUREC/S/481/2025: PG.). The study was classified as minimal-risk research, and the committee granted a formal waiver of individual informed consent. This waiver was justified because the analysis relied exclusively on secondary, non-identifiable, aggregated data from the CEMD, ensuring the confidentiality of all individuals was maintained.

## Results

### National trends in obstetric haemorrhage mortality

**FIGURE 1:**
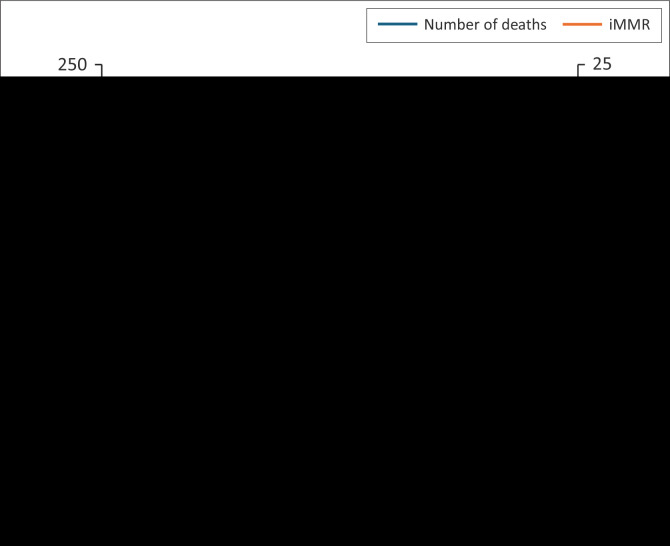
Obstetric haemorrhage mortality in South Africa.^24^

Analysis of the CEMD data revealed significant fluctuations in obstetric haemorrhage mortality during the 2017–2023 period (Figure 1). During the pre-pandemic years (2017–2019), both absolute numbers of deaths and iMMR steadily decreased. The iMMR decreased from 20.1 per 100 000 live births in 2017 to 18.1 live births in 2019. This encouraging trend was disrupted in 2020, with deaths increasing to 200 (iMMR: 19.3) and peaking in 2021 at 237 deaths (iMMR: 23.3), a 28.6% increase from the 2019 baseline.

In accordance with the CEMD triennial reporting structure, this analysis compares provincial data for the periods 2017–2019 and 2020–2022. As detailed in Table 1, the trends in obstetric haemorrhage mortality revealed profound geographical disparities. Three provinces, Eastern Cape, Limpopo and Northern Cape, demonstrated notable resilience, achieving mortality reductions exceeding 25%. The Free State was an outlier of stability, with a negligible change. Conversely, five provinces experienced a deterioration in outcomes. Most notably, the Western Cape, despite maintaining the most favourable absolute mortality rate, registered the most significant relative increase at 33.6%. This was followed by Northwest, Gauteng, KwaZulu-Natal and Mpumalanga, which all reported increased mortality.

**TABLE 1:** Provincial trends in obstetric haemorrhage mortality in South Africa, for the period 2017–2019 versus 2020–2022.^24^

Province	2017–2019 (Deaths per 100 000 live births)	2020–2022 (Deaths per 100 000 live births)	% Change	Trend
Eastern Cape	22.9	17.0	−34.7	Major improvement
Limpopo	28.6	22.7	−26.0	Major improvement
Northern Cape	26.7	20.9	−27.8	Major improvement
Free State	31.4	31.1	−1.0	Marginal improvement
Mpumalanga	26.9	30.4	+11.5	Deterioration
KwaZulu-Natal	12.3	14.7	+16.3	Deterioration
North West	22.9	28.3	+19.1	Deterioration
Gauteng	16.3	19.9	+18.1	Deterioration
Western Cape	7.1	10.7	+33.6	Significant deterioration

*Source*: National Department of Health (South Africa). Saving mothers: National Committee for Confidential Enquiry into Maternal Deaths (NCCEMD) annual report 2023. Pretoria: NDoH, 2023; p. 1–35

## Discussion

This study investigated the impact of the COVID-19 pandemic on obstetric haemorrhage death rates. Substantial quantitative evidence demonstrates that the pandemic significantly worsened obstetric haemorrhage outcomes within SA. The iMMR because of obstetric haemorrhage serves as a crucial metric for evaluating a healthcare system’s capacity to deliver effective emergency obstetric care. The observed data patterns can be analysed within the broader framework of health system challenges and improvement efforts. Prior to the pandemic (2017–2019), a consistent decrease in both the number of deaths and the iMMR indicated that initiatives to enhance maternal care were yielding positive results. This improvement resulted from factors such as better adherence to clinical guidelines for preventing and managing postpartum haemorrhage.^9,10,11^

The COVID-19 pandemic precipitated a substantial increase in obstetric haemorrhage mortality in SA, with the iMMR reaching a peak of 23.3 in 2021. This surge coincided with the Beta and Delta variant waves, a period of high caseload and stringent public health measures.^12^ The increase aligns with global reports on the pandemic’s indirect harms to maternal health,^13^ and is consistent with documented impacts on South African health services.^14^ The decline in maternal outcomes was driven by interconnected systemic disruptions. Overwhelming demand led to the reallocation of critical resources like intensive care unit (ICU) beds and staff away from maternity care,^15^ while lockdowns, transportation issues and patient fear of infection created dangerous delays in seeking care.^16^

Furthermore, supply chain problems likely compromised essential clinical protocols, delaying life-saving interventions for haemorrhage.^17^

The post-peak period (2022–2023) was marked by a substantial and encouraging reversal, with the average obstetric haemorrhage iMMR falling from 21.3 to 16.6, the lowest level recorded in the 7-year period. This significant decline signals a critical recovery of the maternal healthcare system following the extreme stress of the pandemic. The improvement can be largely attributed to the stabilisation of health services, which allowed for the vital reallocation of dedicated ICU beds, consistent blood product supplies, and specialised staff back to core maternity services, thereby mitigating a key administrative avoidable factor exacerbated during the crisis.^18,19^

Concurrently, the lifting of lockdown restrictions and a consequent reduction in the population’s fear of infection were pivotal in restoring timely access to care. As Kassa et al.^20^ note, this shift reversed the dangerous ‘patient-related’ delays that had characterised the earlier pandemic years, ensuring that women presented to facilities sooner for emergency obstetric interventions. Furthermore, the decline suggests that health facilities re-established and reinforced essential clinical protocols for haemorrhage management, which had been disrupted by staff overload and supply chain issues during the peak COVID-19 waves. This return to a more stable operational baseline underscores the health system’s resilience and its capacity to rebound, highlighting the importance of safeguarding essential maternal health services even during public health emergencies to prevent indirect mortality.

The pandemic’s impact on obstetric haemorrhage mortality was geographically heterogeneous across SA, revealing critical variations in provincial health system resilience. While five provinces experienced increased obstetric haemorrhage iMMR, others demonstrated resilience by maintaining or improving outcomes, suggesting pre-existing disparities in systemic strength.^21^ Deterioration in some regions can be partially explained by significant disruptions to essential services. For instance, research from Gauteng province in Tshwane District documented a marked decline in early antenatal care attendance and postnatal visits during lockdowns.^22^ Such interruptions likely led to unmanaged risk factors like anaemia and delays in diagnosing pregnancy complications, thereby exacerbating the severity of haemorrhage events.^23^ The subsequent system-wide recovery observed in 2020–2022, evidenced by a significant decline in mortality in several provinces, indicates a restoration of healthcare functionality. This positive trend may be partly attributable to targeted interventions, such as a national quality improvement programme.^14^ Notably, two of the three provinces where this programme was implemented were among those that showed improved outcomes in the present data, underscoring the potential of such focused efforts to bolster health system resilience post-crisis.

### Strengths and limitations

The strength of this analysis lies in its use of comprehensive national data from the well-established confidential enquiry system in SA, which employs standardised methodology across facilities and provinces. The 7-year timeframe provides an adequate pre-pandemic baseline and allows for the observation of recovery patterns. However, several limitations must be acknowledged. The use of aggregated data prevents more detailed statistical analysis of potential confounding factors and subgroup variations. The absence of detailed clinical data on each case limits the ability to establish direct causal relationships between specific pandemic-related disruptions and individual deaths. Additionally, changes in reporting completeness during the pandemic may have affected data quality, although the confidential enquiry system generally maintains consistent ascertainment.

## Conclusion

The COVID-19 pandemic exposed and amplified pre-existing vulnerabilities in SA’s maternal healthcare system, with particularly severe consequences for the management of obstetric haemorrhage. The significant increase in haemorrhage-related mortality during the peak pandemic years represents a reversal of previous progress and underscores the fragility of emergency obstetric services in the face of health system shocks. The confidential enquiry process has proven invaluable in documenting these trends. The decline in haemorrhage mortality in the post-peak period suggests that system learning and adaptation have occurred, potentially through the implementation of recommendations generated by the enquiry process.

Moving forward, building resilient maternal health systems requires deliberate planning to protect essential obstetric services during future emergencies. This includes ensuring resource prioritisation for time-critical conditions like haemorrhage, developing adapted clinical protocols for crisis situations, and strengthening community trust in the health system’s ability to provide safe care during emergencies. The lessons from SA’s experience with COVID-19 and obstetric haemorrhage mortality offer valuable insights for other middle-income countries seeking to strengthen their maternal healthcare systems against future shocks. Further research should focus on understanding the factors that contributed to the successful reduction in haemorrhage mortality in the post-peak period, as these may offer replicable strategies for enhancing health system resilience globally.
